# Dry-Etched Oxide Templates Enable Scalable and Waste-Free Synthesis of Graphene-Based Aeromaterials

**DOI:** 10.1021/acsaenm.6c00181

**Published:** 2026-05-06

**Authors:** Morten Möller, K. Meurisch, J.-O. Stern, A. Reimers, F. Schütt, Sören Kaps, Rainer Adelung

**Affiliations:** Functional Nanomaterials, Department of Materials Science, Kiel University, Kaiserstr. 2, 24143 Kiel, Germany

**Keywords:** aerographene, graphene aeromaterials, dry-etching processes, forming-gas etching, tetrapodal ZnO, structure tailoring, scalable synthesis, lightweight materials, sensor technology, filtration, wet-chemical assembly

## Abstract

Template-assisted synthesis is widely used to fabricate ultralight carbon aeromaterials, yet conventional wet-chemical template removal requires multistep solvent exchange and critical point drying, which limits scalability, reproducibility, and environmental efficiency. Here, we introduce a dry-chemical template-removal process using forming gas (H_2_/N_2_) as a liquid-free, furnace-based alternative and evaluate it using graphene-coated tetrapodal ZnO as a representative oxide-templated aeromaterial system. Thermogravimetric and calorimetric analyses identify temperature-dependent reaction regimes, including hydrogen-driven reduction, vapor-transport-limited zinc evaporation, and carbothermic reduction at elevated temperatures, which collectively govern pore opening and wall thinning within the graphene shell. Etching temperatures of 800–1000 °C produce architectures with high structural integrity, whereas higher temperatures further increase porosity but introduce additional defects. Mechanical and mechanoelectrical testing show that dry-etched aeromaterials retain the elastic compliance of wet-etched references while exhibiting up to 4-fold higher electrical conductivity, attributed to thermally enhanced interflake coupling and removal of residual adsorbates. Because zinc is released in vapor form, the method also offers prospects for material recovery and circularity. Overall, gas-phase template removal provides a scalable and environmentally efficient pathway for producing high-performance graphene-based aeromaterials.

## Graphical abstract

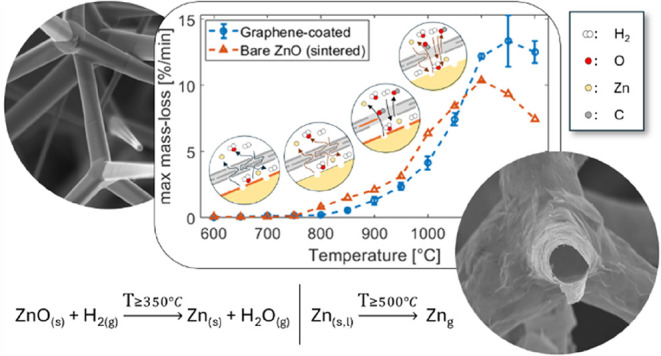

## Introduction

The advancement of functional carbon nanomaterials continues to face two fundamental, closely related challenges: bridging the gap between their nanoscale nature and macroscopic applicability, and developing reliable methods to assemble inherently one- or two-dimensional (1D/2D) building blocks, such as carbon nanotubes or graphene, into structurally stable, functionally integrated three-dimensional (3D) architectures.^2–7^ Hierarchically structured 3D carbon-based aerogels and foams, especially those containing graphene or carbon nanotube (CNT) components, represent a promising material platform for this purpose. They offer an exceptional combination of ultralow density, high specific surface area, and high electrical conductivity,^2,3,8–10^ making them attractive for use in sensors,^11–13^ energy storage,^14–16^ filtration,^17^ and lightweight structural components.^9,18–22^ Within this broader class of carbon aeromaterials, graphene-based hollow microtube or cellular networks, obtained by conformally coating sacrificial oxide scaffolds with exfoliated graphene or other carbon precursors, form a particularly compelling subclass. Their extremely low density, mechanical flexibility, high thermal stability, and outstanding electrical conductivity make them highly suitable for piezoresistive sensing,^11^ high-efficiency microfiltration,^17^ and soft actuation systems.^23–25^

A widely used strategy for producing such three-dimensional carbon aerogels and graphene-based architectures is the template-assisted synthesis using sacrificial inorganic scaffolds. Metal oxides such as ZnO, MgO, MnO_2_, NiO, or SiO_2_ can be shaped into well-defined porous networks, infiltrated with carbon precursors or graphene dispersions, and subsequently removed to yield free-standing hollow or cellular carbon frameworks.^1,26–28^ This approach has been adopted across diverse research fields, including energy storage, catalysis, sensing, and membrane technologies, due to its ability to replicate complex 3D architectures with high structural fidelity.^9,29–34^

Over the past decade, wet-chemical template removal has proven to be a highly effective and widely adopted strategy for producing oxide-templated carbon aerogels with excellent structural fidelity. Through carefully optimized etching protocols, solvent-exchange procedures, and critical point drying (CPD), numerous studies have demonstrated that even extremely delicate carbon architectures can be preserved with remarkable precision.^1,35^ These methods have enabled a broad range of functional aeromaterials and remain a cornerstone of template-assisted synthesis. Nevertheless, despite their demonstrated success, wet-chemical template removal inherently involves multistep solvent handling and drying operations, which impose practical limitations on throughput, reproducibility, and scalable processing. The reliance on CPD or freeze-drying, while indispensable for mitigating capillary forces, introduces additional complexity, potential for structural damage during handling and increases the environmental burden. Freeze-drying can offer advantages in certain cases, but the risk of ice-crystal-induced deformation remains a known challenge for ultralight architectures.^36^ These constraints do not detract from the effectiveness of established wet-chemical techniques; rather, they motivate the exploration of complementary, alternative pathways that may further expand the design and processing space for next-generation aeromaterials.

Among the broad family of ultralight carbon aeromaterials derived from sacrificial oxide templates, graphene-based variants are particularly sensitive to such processing limitations. Their characteristic hollow tubular or cellular microstructures, formed by conformal coatings of exfoliated graphene or other carbon precursors around ZnO, MgO, or similar oxide networks, enable extremely low densities and high electrical conductivities, but also render them highly susceptible to capillary-induced deformation and mechanical damage during wet-chemical template removal. As a result, reliable preservation of these fragile architectures often requires carefully orchestrated multistep drying sequences, which can constrain scalability and overall process robustness.

Dry-chemical template removal provides a fundamentally different processing route that eliminates liquid-phase handling, simplifies workflow complexity, and enables scalability to significantly larger sample formats (Figure S1). Gas-phase etching approaches can, in principle, be applied to a broad range of reducible or volatile metal oxides, offering a compelling alternative to classical wet-chemical pathways while maintaining the integrity of fragile carbon architectures.^37–39^ Nevertheless, systematic studies on dry removal of oxide templates, especially in the context of graphene-based aerogels, remain scarce.

In this study, we introduce a dry-chemical etching process using forming gas (H_2_/N_2_) at elevated temperatures to remove ZnO templates as a model system. Beyond reducing process time and enhancing scalability (Figure 1), the method enables controlled tuning of microstructural features such as wall thickness, porosity, and connectivity. These parameters directly influence mechanical stiffness and electrical conductivity, thereby providing a systematic pathway toward scalable, application-specific design of 3D carbon aeromaterials.

**Figure 1:**
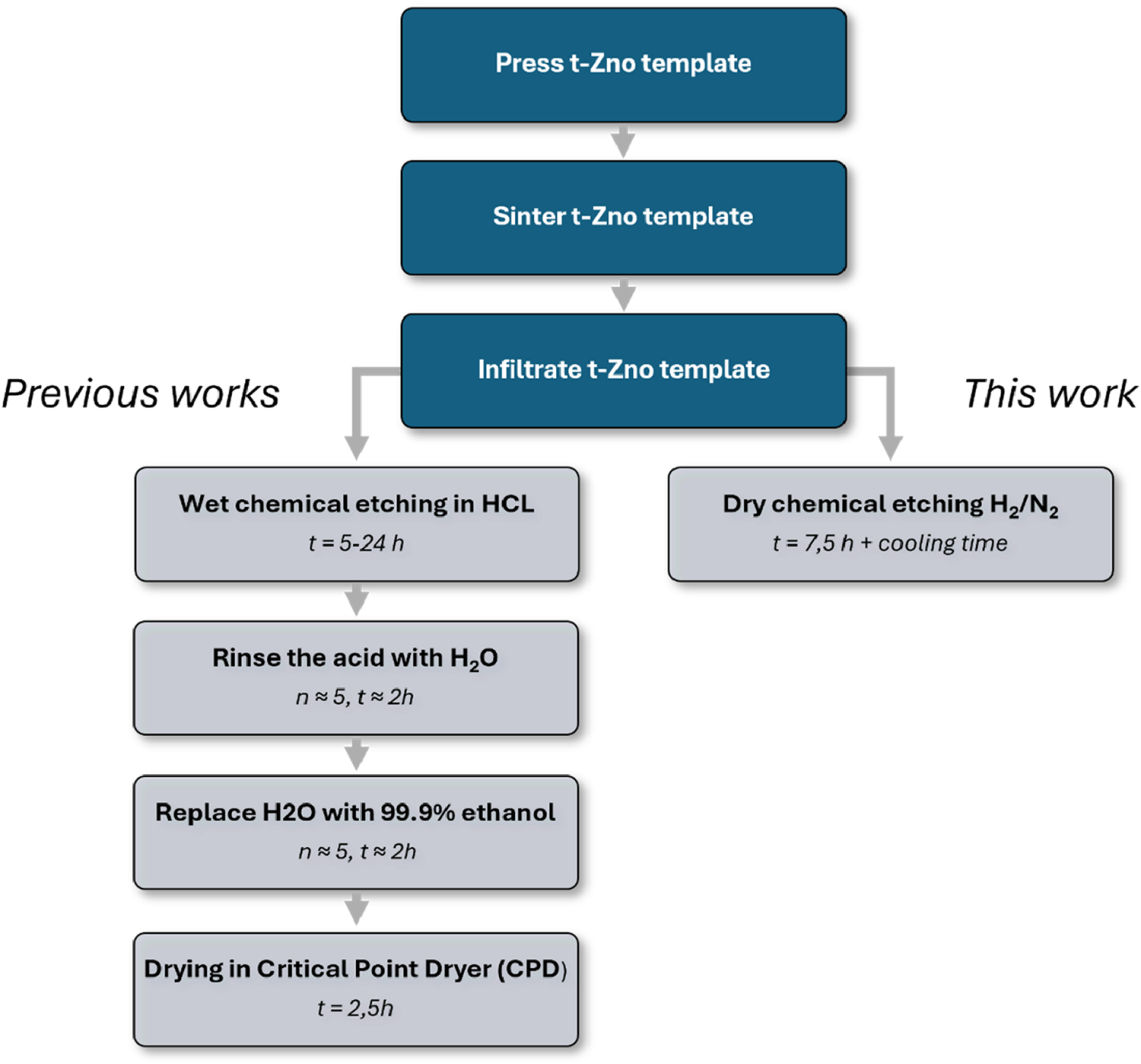
Comparison of wet and dry chemical ZnO etching processes. Comparison of the individual process steps of the etching process according to Rasch et al.^1^ *and the dry chemical etching process presented here*.

## Experimental Section

### Sample Preparation

The tetrapodal zinc oxide (t-ZnO) microparticles were in-house synthesized by the flame transport synthesis method described by Mishra et al.^30^ The material (Figure 2) consists of tetrapod-shaped ZnO microparticles, i.e., three-dimensionally extended microstructures.

**Figure 2:**
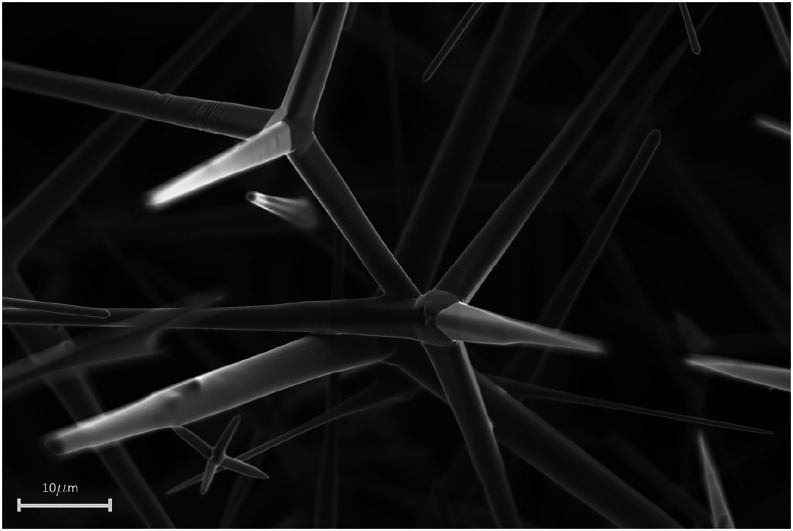
SEM image of the tetrapodal ZnO (t-ZnO) template before graphene infiltration. The template forms an open porous network with a density of **0.3 g cm** ^**–3**^, corresponding to a porosity of approximately **94%**.

These microparticles were subsequently pressed and sintered into a porous template according to the procedure described by Rasch et al.,^1^ resulting in a template density of 0.3 g cm^–3^. For coating, the templates were infiltrated five times with a 1.4 mg mL^–1^ exfoliated graphene dispersion provided by Sixonia (Dresden, Germany). According to manufacturer-provided specifications, the dispersion is based on flakes with a mean lateral size of approximately 0.5–1 μm, a thickness below 3 nm, and 1–10 graphene layers.

Between successive infiltration steps, the coated samples were dried at 50 °C for 4 h to allow solvent evaporation and deposition of the graphene flakes onto the template surface. In the final step, the ZnO templates were removed either by dry-chemical etching in a furnace with a protective-gas retort (Carbolite Gero 1100), in a thermogravimetric analysis device (TA Instruments SDT 650; Figure 3), or by a wet-chemical reference procedure adapted from Rasch et al.^1^ In the latter case, the ZnO was Subsequently etched in 1 M HCl for 24 h, washed with water and absolute ethanol, and dried by critical point drying (CPD; EMS 3000).

**Figure 3:**
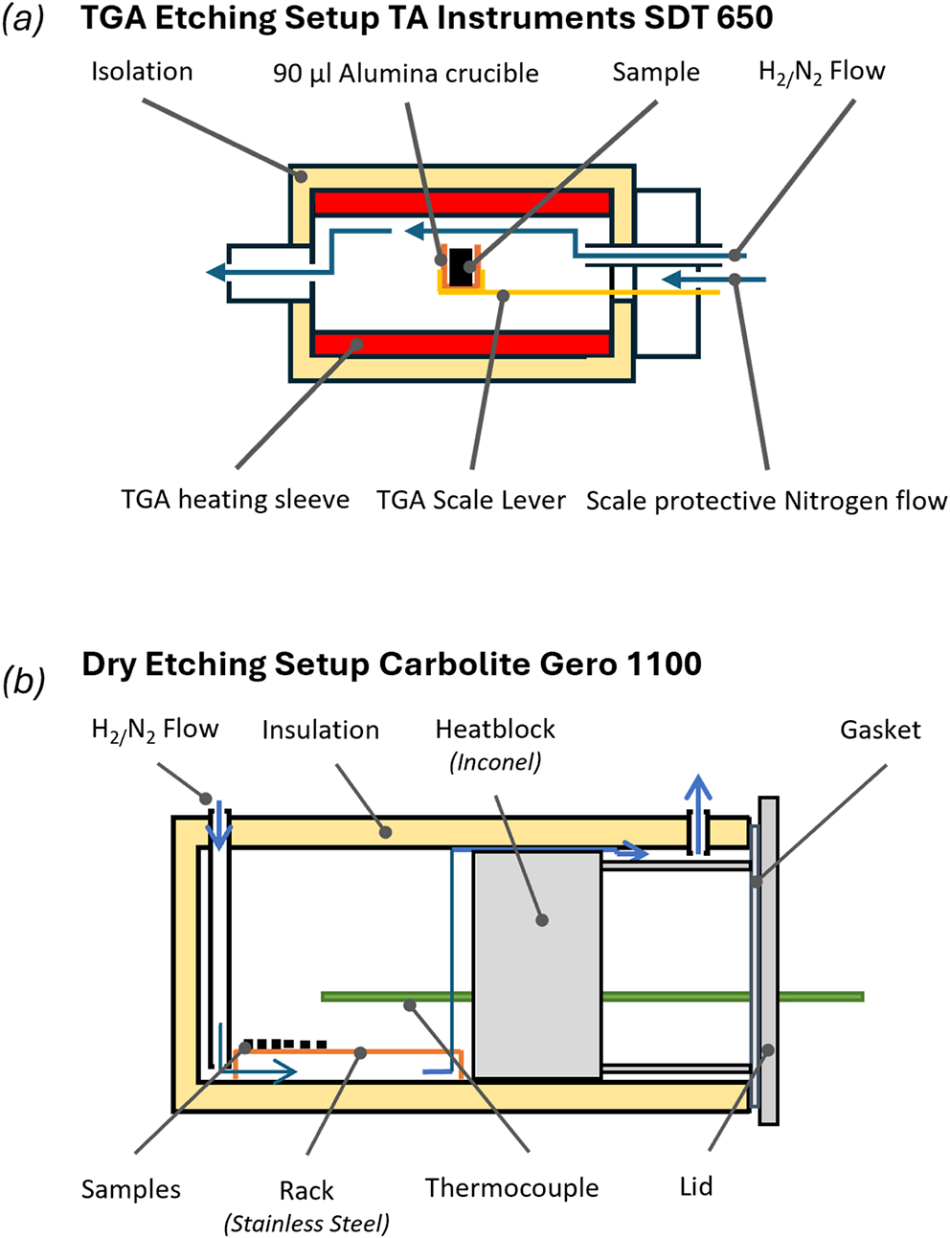
Schematic diagrams of the experimental setups with the Carbolite Gero 1100 furnace and a protective gas retort (a) and the cross-section of the reaction chamber of the TGA SDT 650 from TA Instruments (b).

Both wet-chemical and dry-chemical template removal routes were implemented to allow a direct and reproducible comparison between the established CPD-based approach and the newly introduced gas-phase process. For the present study, a matched wet-chemical reference route was used to enable direct comparison under consistent experimental conditions. All samples were synthesized under identical precursor and templating conditions to ensure that any subsequent differences arise solely from the respective template-removal strategy.

The forming gas flow (3 L h^–1^) was initiated at room temperature and maintained throughout the heating process. The selected flow rate was based on preliminary optimization experiments for the specific furnace/retort configuration used in this study. The heating rate of 8 °C min^–1^ was given by the technical constraints of the protective-gas retort of the Carbolite Gero 1100. Due to this moderate heating rate, sufficient purging time was ensured during the ramp phase. It is widely accepted that graphene remains stable in ambient atmosphere up to approximately 350 °C, providing a conservative safety margin below the typical onset of oxidative degradation (≥400–450 °C). Therefore, forming gas flushing was considered effective throughout the entire heating period up to this temperature. Etching temperatures were varied from 600 to 1200 °C in 100 °C increments. After a holding period of 300 min, passive cooling to 200 °C was initiated. When 200 °C was reached, the forming gas purging was stopped and the process was defined as complete. At each temperature stage, 6 cylindrical samples were placed in the furnace: three for characterization in the SEM and three each for mechanical and mechano-electrical characterization.

To investigate the etching process in more detail, TGA experiments were conducted. Initially, the TGA crucible was flushed with forming gas at an externally controlled volume flow rate of 120–135 mL min^–1^ for 20 min at room temperature. The temperature was then increased at a rate of 25 °C min^–1^ to the specified target temperature. This varied in the range from 600 to 1200 °C, with the temperatures being increased in 50 °C increments for each experiment. Once the target temperature had been reached, a holding phase of 300 min followed. The etching process was completed with a programmed cooling rate of 50 °C min^–1^. This cooling rate could be maintained at higher temperatures, while it decreased as the temperature dropped. Three etching experiments were carried out for each target temperature.

Table 1 lists the respective furnaces, forming gas compositions and sample geometries for the different characterization methods.

**Table 1:** Overview of the Samples Produced

characterization	furnace geometry	forming gas	cylinder geometry
SEM	protective gas furnace	10% H_2_, 90% N_2_	*h* = 2 mm, *d* = 12 mm
micromanipulator	protective gas furnace	10% H_2_, 90% N_2_	*h* = 6 mm, *d* = 6 mm
effective density	protective gas furnace	10% H_2_, 90% N_2_	*h* = 6 mm, *d* = 6 mm
thermogravimetry	TGA crucible	5% H_2_, 95% N_2_	*h* = 3 mm, *d* = 4 mm

### Characterization

This characterization framework enables a systematic assessment of how dry-chemical template removal affects process reproducibility and scalability compared to the wet-chemical CPD route, examined and demonstrated here using the graphene-coated t-ZnO–derived aeromaterial as a representative model system for oxide-templated 3D carbon architectures.

SEM images were taken of dry-etched aerographene samples with a diameter of 12 mm and a height of 2 mm. For this purpose, a scanning electron microscope (SEM) (Zeiss Supra 55VP) with an in-lens detector and acceleration voltages of 3 and 10 kV was used.

To calculate the effective aerographene density, the samples were weighed after each process step (template material, template sintering, template infiltration, template etching) and the geometry (height: h, diameter: d) was determined by means of photographic documentation and digital evaluation. Volumes were calculated accordingly. The uncertainty of the effective density was calculated from the uncertainties of the measured sample mass, diameter, and mean height. The effective densities given in this work represent the mean value of a total of 5 wet-etched samples and 3 dry-etched samples at a specific temperature.

The thermogravimetric measurements were performed on a TA Instruments device (SDT 650). To achieve consistent results, care was taken to always place the samples centrally and upright in the crucible. Three experiments were performed for each target temperature to ensure the reproducibility of the results. The results presented in Figure 6 and corresponding discussion are based on the mean values of the respective measurements. It should be noted that the forming gas composition, furnace geometry and sample dimensions for the thermogravimetric measurements could not be kept the same as in the protective gas retort (see Figure 3 and Table 1). Nonetheless, qualitative statements about the dry etching process could be derived from this experiment. In particular, the TGA was operated with 5% H_2_ in N_2_, whereas the furnace experiments were conducted with 10% H_2_ in N_2_. As both conditions provide hydrogen in large stoichiometric excess, the TGA evaluation is used here primarily for qualitative identification of reaction regimes. Any quantitative differences in apparent kinetics may also reflect differences in transport conditions, gas access, and product removal between both setups.

Three cylindrical samples per temperature level were prepared for mechanical and mechanoelectrical characterization. Three additional samples were etched using the conventional wet-chemical process and served as references. All samples were cyclically compressed using a micromanipulator (Märzhäuser Wetzlar GmbH & CO. KG HS6 3 Achsen – 0.4 mm) on a force transducer (burster 8523–5050). Electrical contact was established via gold-coated contacts. Mechanical and electrical contact between the sample and the measuring electrode was established via gold-coated contacts using a force-controlled contacting procedure (Figure 4a). To reduce the influence of minor sample-to-sample variations in macroscopic geometry and end-face alignment, the manipulator arm was lowered until a defined force threshold of 0.02 N was reached, as measured by the force transducer described above. Each sample was first compressed cyclically five times by 5% of its original height, followed by a single compression to 30%. The compression was performed with a programmed amplitude, period duration, and phase (see Figure 4b). The measurement data included force (Figure 4c), displacement, voltage, current, and time, from which electrical resistance (Figure 4d), compression, and modulus of elasticity were calculated. The conductivity values reported in this work represent apparent conductivities of the porous macroscopic samples, derived from the measured resistance and sample geometry, rather than intrinsic bulk conductivities of dense graphene material.

**Figure 4:**
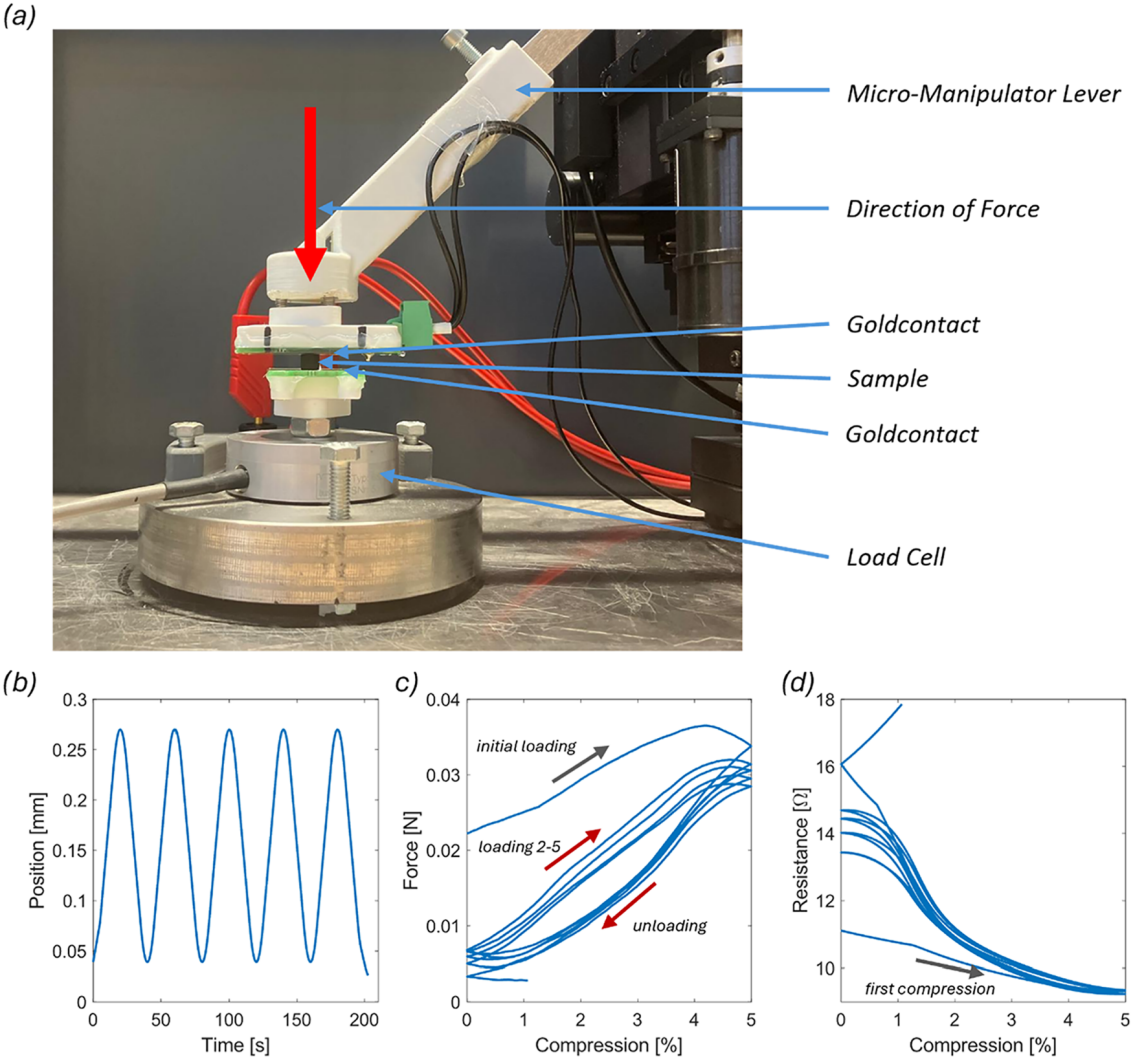
(a) Micromanipulator setup as a close-up shows a cylindrical sample on PCB surface. PCB is mounted on the Loadcell and the Micromanipulator lever. (b) Example measurement cycle of 5 × 5% compression of a 900 °C sample. (c) Example loadcycle for a 5 × 5% compression experiment on a 900 °C sample. (d) Example electrical resistance for a 5 × 5% compression experiment on a 900 °C sample.

Due to the viscoelastic and porous nature of the samples, the apparent elastic modulus was calculated from the slope of the linear region of the force–displacement curve during compression. A linear fit was applied to the loading segment between 12.5 and 87.5% of the maximum force.^9^ Based on the sample’s initial length and cross-sectional area, the modulus was derived using Hooke’s law. All values were averaged per temperature group across three measurements. Where replicate-based averaged data are shown, values are reported as mean ± standard deviation (SD).

## Results and Discussion

Initially, a macroscopic and microscopic investigation of the morphology of the etched aerographene samples was conducted. Thin graphene layers become electron-transparent at higher accelerating voltages, rendering SEM imaging particularly suitable for assessing the degree of ZnO template removal after dry-chemical etching at a given process temperature. Figure S2 shows images of a sample that was incompletely etched after 5 h at 600 °C. It can be observed that the dry-chemical etching process removes ZnO along the tetrapod arms of the template from all sides. The constant distance between the graphene shell and the ZnO core indicates a uniform diffusion of hydrogen molecules through the thin layer of overlapping, van-der-Waals-bonded graphene flakes.^8^ The enhanced removal at tetrapod tips and thinner tetrapod arms can, under the assumption of a homogeneous graphene coating,^1^ be explained by the locally increased surface-to-volume ratio of the ZnO.

Figure 5 illustrates the temperature-dependent structural changes across multiple length scales by showing photographs of the cylindrical aerographene samples after ZnO etching in the protective-gas furnace, combined with SEM images of samples etched in the same process. With increasing etching temperature, macroscopic deviations from the original cylindrical template shape and, on the microscopic scale, progressive wall thinning and microstructural irregularities of the graphene shell become apparent. While such microscale defects and wall thinning become more pronounced at elevated temperatures, these features do not necessarily compromise performance; instead, they can facilitate improved internal accessibility and electrical connectivity within the resulting carbon architecture.

**Figure 5:**
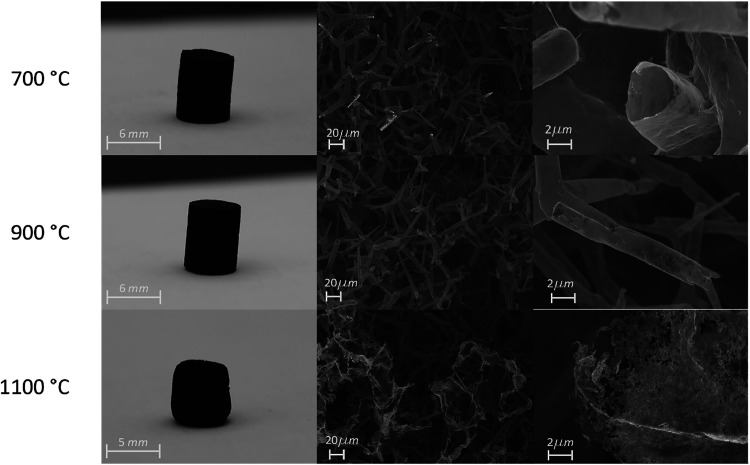
Photos and SEM images of cylindrical Ø6 mm × 6 mm high aerographene samples corresponding to the synthesis temperature with associated SEM images assigned to each temperature in rows. (For more synthesis temperatures see Figure S3).

This increase in defects is reflected in the effective densities of the dry-chemically etched aerographene (cf. Figure S4). For final characterization of the fabricated aerographene, the effective density ρ_eff_ was obtained from the postetch mass and the measured sample volume. The porosity was then calculated relative to bulk graphite ρ_graphite_ = 2.26 g cm^–3^ according to eq 1.

1 $$\phi \;\left(\%\right)=100\times \left(1-\frac{\rho_{eff}}{\rho_{\mathrm{graphite}}}\right)$$

Across 800–1100 °C, ρ_eff_ remained far below the graphite reference, confirming highly porous structures. A continuous decrease in ρ_eff_ with increasing temperature was observed, indicating either enhanced removal of residual material or expansion of the pore network. Particularly striking was the reduction in effective density between 800 and 1100 °C by 7.6%. In contrast, the wet-etched reference samples exhibited effective densities that were approximately 70–80% higher than those of the dry-etched aerographene. With increasing temperature, porosity showed a slight upward trend; the porosity increased from about 99.76% at 800 °C to 99.78% at 1100 °C, corresponding to an absolute increase of approximately 0.02 percentage points. This seemingly small absolute change in porosity is consistent with the fact that the aeromaterial already exhibits an ultrahigh baseline porosity (>99.7%), such that even minor geometric alterations, including wall thinning or pore opening, result in comparatively large relative changes in effective density. In addition, the porosity values are derived from the postetch mass and the measured sample volume; therefore, the observed shrinkage must be considered together with the simultaneous graphene mass loss during high-temperature treatment. While the wet-chemically etched reference samples displayed comparable porosities, their values were generally lower.

Also, the geometrical evolution during the processing steps was investigated and is shown in Figure S5. It can be concluded that the shrinkage in height is more pronounced than the shrinkage in diameter. The resulting decrease in overall volume is presented in Figure S6 and indicates that between 800 and 1000 °C an average volume shrinkage of approximately 12.5% (±7.5%) occurs. This shrinkage is not attributed solely to ZnO removal, but is interpreted as a combined consequence of template removal and temperature-dependent modification of the graphene framework itself. In this context, graphene has been reported to exhibit negative thermal expansion,^40,41^ while thermally induced reorganization of flake-based graphene networks may further contribute to macroscopic shrinkage.^42^ Compared to wet-chemical template removal, the dry-chemical route leads to substantially reduced macroscopic deformation, indicating that the absence of liquid-phase capillary forces preserves the overall shape more effectively. This reduced geometric distortion highlights the mechanical gentleness of the gas-phase process and underscores its suitability for scalable batch processing of fragile aeromaterial architectures. These temperature-dependent morphological trends constitute the structural basis for interpreting the mechanical and electrical behaviors discussed in the subsequent sections.

To quantify graphene retention during template removal, the introduced graphene mass was determined from the mass difference between the infiltrated and sintered states, and the amount remaining after etching was referenced to this initial value. Supplementary Figure S7 displays the relative graphene mass retained after etching as a function of process temperature. All samples exhibited at least a 25% mass loss. The wet-chemical reference process yielded the lowest average loss (25%), whereas the dry-chemically etched samples (800–1000 °C) consistently showed a loss of approximately 45%. Notably, the variance in graphene retention decreased with increasing temperature, suggesting either enhanced stabilization of the graphene network or a temperature-dependent modification of pore morphology.

To elucidate the underlying etching mechanism, thermogravimetric analysis (TGA) coupled with differential scanning calorimetry (DSC) was performed using a TGA/DSC 650 system (TA Instruments). The objective was to obtain temperature-dependent mass-loss and heat-flow profiles and to derive a theoretical model of the chemical reaction processes. In addition to characterizing the specific material system examined here, the TGA analysis allows identification of general temperature-dependent reaction features that are relevant for hydrogen-assisted removal of reducible oxide templates. (According to the TA Instruments convention, endothermic events are plotted downward, exothermic upward.)

Figure 6a displays the average mass-loss over process time at different temperatures. The data clearly demonstrate that the removal of the ZnO scaffold structures accelerates significantly with increasing temperature, resulting in complete mass loss within progressively shorter time intervals. Although the thermal boundary conditions in the TGA differ from those in the protective-gas retort, the resulting mass-loss signatures reflect intrinsic reaction kinetics rather than apparatus-specific artifacts, and therefore allow robust qualitative identification of reaction regimes that are relevant also for large-scale furnace etching.

**Figure 6:**
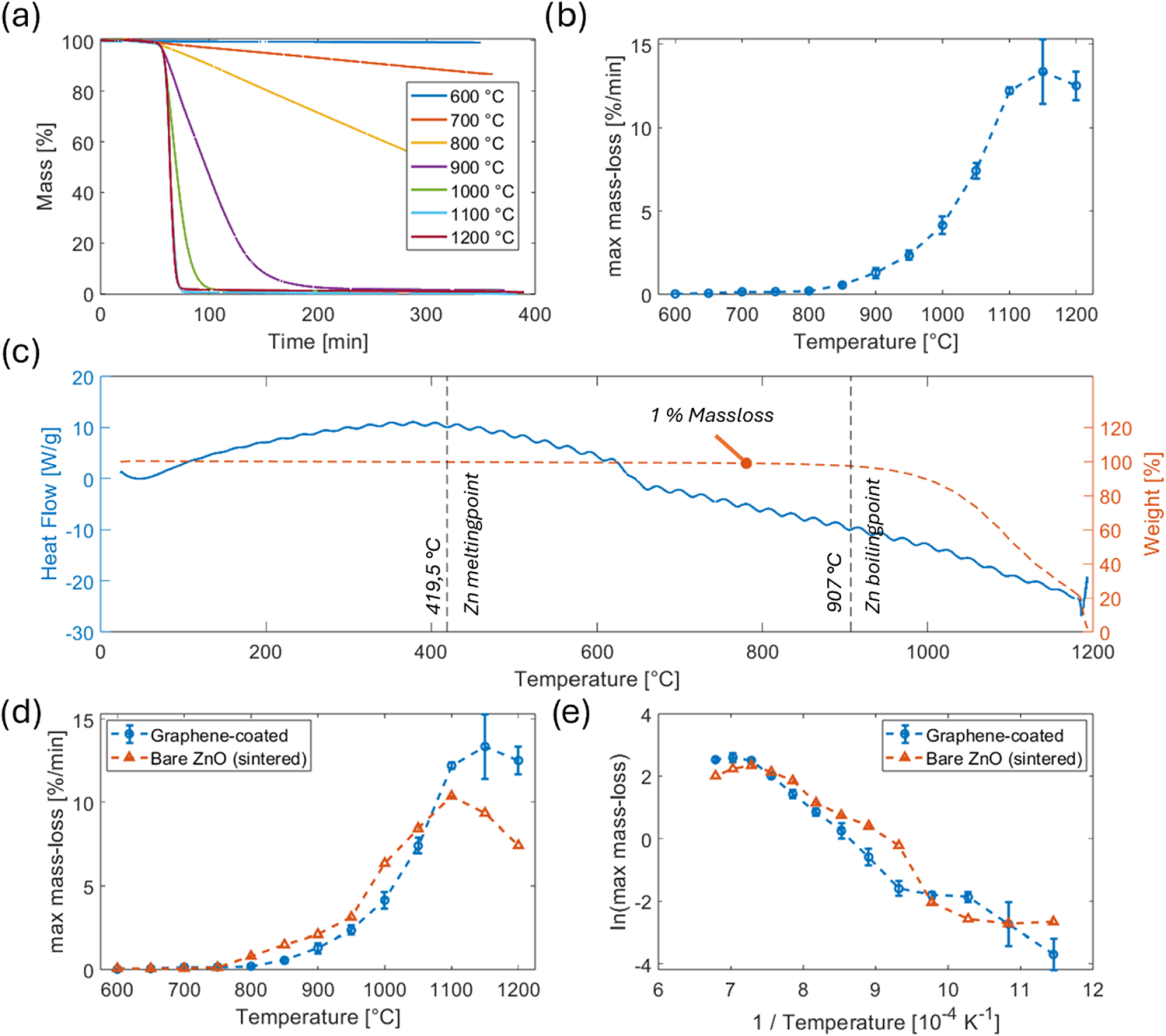
TGA experiment analysis: (a) Mass in % over time at different temperatures. (b) Maximum averaged mass-loss at all tested temperatures. (c) Heat flow plot of a 1200 °C Experiment. (d) Maximum mass-loss of graphene-coated and bare ZnO templates as a function of temperature. (e) Arrhenius-type plot of the same data, used for qualitative identification of distinct regime transitions, with graphene-coated samples exhibiting two slope changes around 700 and 800 °C. Data in panels (a and b) are based on mean values of three independent experiments per temperature (*n* = 3). Error bars represent standard deviation.. In panel (d), graphene-coated templates were measured with *n* = 3, whereas the bare ZnO reference is shown for *n* = 1. Dashed connecting lines in panels (a, d, e) are provided for visual clarity only and do not indicate further data points.

Figure 6b presents the maximum mass-loss as a function of temperature. As expected, the loss rates increase markedly with rising temperature, reaching a maximum around 1150 °C. Beyond this point, stronger fluctuations in mass-loss become apparent, indicating an increasing influence of transport phenomena (gas access and product removal) as well as secondary reaction pathways.^43^

This highlights the dynamic nature of the etching process, in which higher temperatures promote rapid and efficient template removal, while moderate temperatures are more favorable for maintaining reproducibility and process control.

The DSC analysis (Figure 6c) reveals several clearly distinguishable thermal events; we propose the following mechanism to interpret the observed thermal events: In the temperature range between approximately 22 and 163 °C, desorption of weakly bound species occurs, accompanied by a nearly constant mass, which is attributed to physisorbed water or residual solvents. This initial desorption regime is characteristic for most oxide-templated aeromaterials, as weakly bound volatiles are universally removed before reduction or evaporation processes dominate.^44^

The exothermic event observed between approximately 163 and 400 °C in the DSC signal, which occurs without a measurable mass loss, is interpreted as a potential indication of ZnO reduction in a hydrogen atmosphere. This interpretation is supported by thermodynamic calculations: the reaction enthalpy for the reduction of ZnO to elemental zinc by molecular hydrogen is approximately −113 kJ mol^–1^ at 350 °C, confirming the exothermic nature of the process. According to Natha et al.,^37^ significant ZnO reduction in hydrogen begins at around 350 °C, marking the onset of elemental zinc formation in near-surface regions. The absence of a concurrent mass loss within this temperature range is consistent with expectations, as the reaction products (Zn and H_2_O) may initially remain adsorbed on the surface or structurally incorporated and thus not immediately detectable as a mass change. Similar kinetic signatures and microstructural transformations during ZnO reduction in hydrogen atmospheres have been reported in the literature.^37^

We hypothesize that, at around 419.5 °C,^45^ the initially formed elemental zinc at the outer surface of the ZnO framework, produced by early stage reduction, is present in the liquid state. At approximately 500 °C,^37^ the equilibrium vapor pressure of zinc reaches values in the millibar range. Under the applied forming gas flow, zinc vapor is continuously removed from the surface by forced convection, keeping the local zinc partial pressure below equilibrium and thereby sustaining evaporation via a persistent concentration gradient.

A pronounced transition in thermal behavior is observed around 660 °C, marked by a distinct step in the DSC signal. This may suggest a regime change, where the reduction of ZnO to metallic Zn by H_2_ possibly dominates below this temperature, while zinc evaporation could play a progressively more significant role in the mass loss above 660 °C.

In the range between 850 and 1000 °C, a clearly measurable mass loss is observed for the first time, accompanied by a steadily increasing endothermic heat uptake (cf. 1% Massloss Figure 6c). This suggests ongoing zinc evaporation, which may become limited by the transport of vapor-phase products. At temperatures above 1000 °C, both the mass loss and the endothermic heatflow of the process continue to increase.

Figure 6d compares the maximum mass loss as a function of process temperature for two sample types: uncoated bare t-ZnO samples (*n* = 1) and graphene-coated t-ZnO templates (*n* = 3) as already shown in Figure 6b. Up to approximately 700 °C, the etching rates of both sample types are nearly identical, suggesting a shared reaction mechanism that is not yet affected by the presence of the graphene layer. It is plausible that the initial reduction of ZnO by hydrogen at the exposed surface dominates in this temperature regime.

Above 700 °C, the uncoated sample shows a steeper increase in etching rate compared to the coated templates. The difference in mass-loss rate remains positive throughout this range, indicating that ZnO initially etches overall faster than ZnO+C. Around 1100 °C, the uncoated sample reaches its maximum mass-loss rate. At the same time, the coated templates display a more gradual rise until their own maximum is reached.

Notably, the relative behavior reverses above ∼1050 °C, where the graphene-coated templates begin to etch faster than the uncoated reference, suggesting a temperature-dependent shift in the rate-limiting mechanism, particularly regarding product release and transport limitations. We hypothesize that this behavior may be influenced by the onset of the reaction of ZnO and CO to Zn and CO_2_ (see **(2**)^46^ which could become increasingly relevant at higher temperatures and alter the local reaction environment beneath the graphene layer. The initially lower etching rates of the graphene-coated templates (up to ∼1050 °C) can be explained by the assumption that the graphene layer acts as a diffusion barrier for zinc vapor, thus hindering the removal of reaction products.

Figure 6e displays the corresponding Arrhenius-type plot of maximum mass-loss rates, revealing two distinct reaction regimes. For uncoated ZnO, the curve begins with a shallow slope, indicating transport limitation in the lower temperature range, likely due to restricted access of the reducing gas or delayed removal of volatile products. Above 700 °C, a significant increase in slope is observed, pointing to a transition into a reaction-controlled regime, possibly resulting from the overcoming of initial diffusion barriers and the rising vapor pressure of Zn.

In contrast, the graphene-coated templates exhibit a more complex behavior: their Arrhenius curve displays two pronounced slope changes, at approximately 700 and 800 °C. These transitions indicate regime shifts in the dominant reaction mechanism, such as transitions from reaction-controlled to transport-controlled stages. They may also mark the onset of additional processes, including carbothermic reduction or enhanced evaporation driven by pore formation within the graphene shell.

Figure 7 summarizes the developed hypothesis of the thermal reaction behavior during the removal of the ZnO scaffold beneath the graphene coating, presented in an Arrhenius-type plot with a schematic assignment of reaction phases. This representation is complemented by ex-situ SEM images of aerographene samples etched at various temperatures, illustrating the structural evolution associated with each phase. The thermal etching process can be subdivided into four distinct regimes. In Phase 1 (up to ∼700 °C), the process is reaction-controlled as it is dominated by the hydrogen reduction of ZnO to metallic zinc, following the reaction:

2 $${Z\mathrm{nO}}_{\left(s\right)}+H_{2\left(g\right)}\to {Zn}_{\left(s,l\right)}+H_{2}O_{\left(g\right)}$$

**Figure 7:**
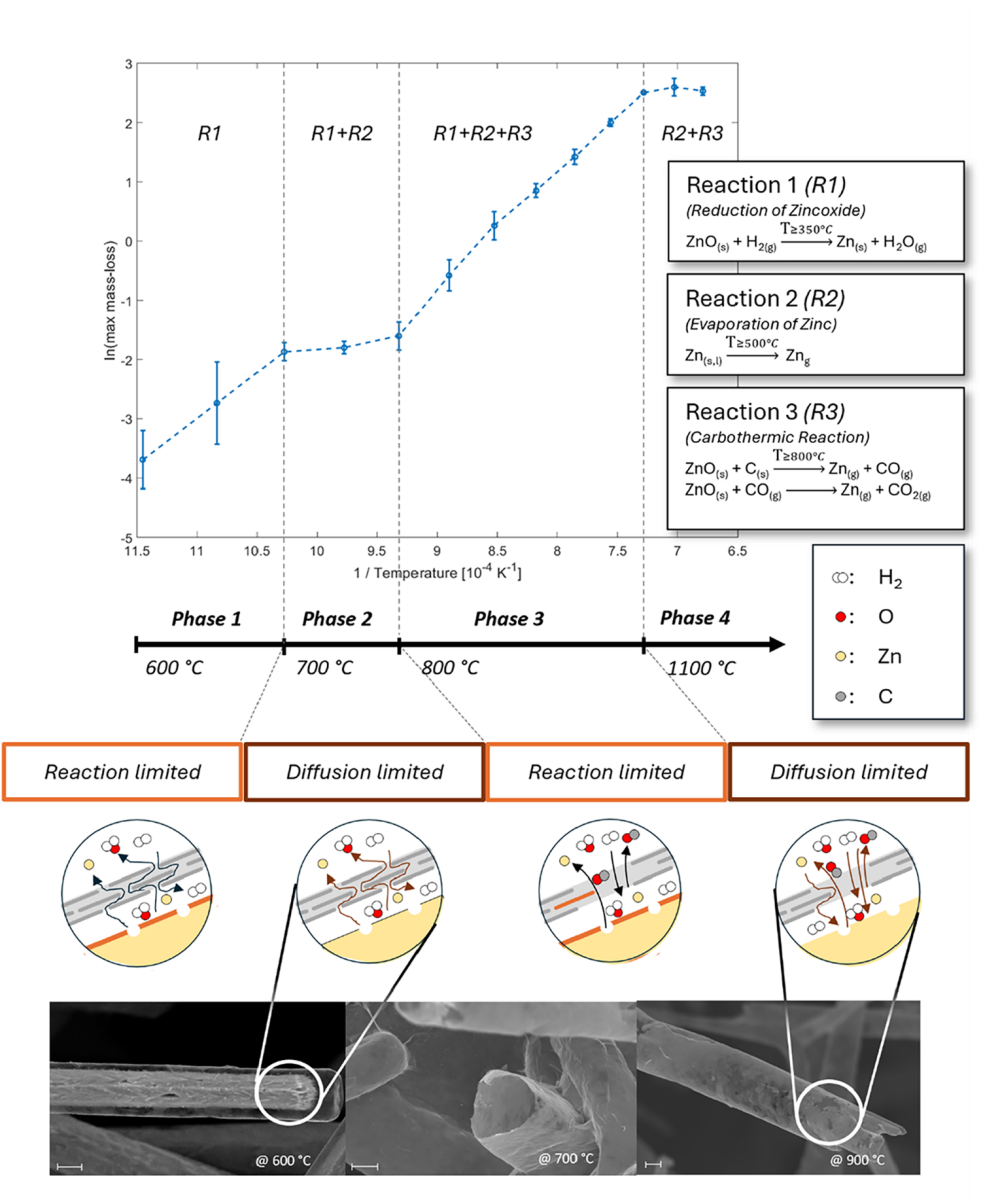
Phase model of the ZnO etching process under graphene, based on Arrhenius analysis. Four temperature-dependent regimes are distinguished, supported by ex situ SEM images of aerographene samples etched at representative temperatures. In the schematic sketches, the orange line indicates the locally dominant reaction front, while the arrows in the diffusion-limited regimes indicate the prevailing transport limitation. The gray shell represents graphene and the yellow region ZnO. Scalebars equal 2 μm. Values in this figure are reported as mean ± SD (*n* = 3).

In Phase 2 (beginning around 700 °C), the increasing vapor pressure of zinc leads to a transport-limited regime, where the graphene layer begins to act as a diffusion barrier, restricting the diffusion of reaction products and thereby limiting the overall process rate. In Phase 3 (from ∼800 °C), an additional carbothermic reduction reaction becomes active.^15^

3 $$C_{\left(s\right)}+{\mathrm{ZnO}}_{\left(s\right)}\to {\mathrm{Zn}}_{\left(g\right)}+{\mathrm{CO}}_{\left(g\right)}$$

4 $${\mathrm{CO}}_{\left(g\right)}+{\mathrm{ZnO}}_{\left(s\right)}\to {\mathrm{Zn}}_{\left(g\right)}+{\mathrm{CO}}_{2\left(g\right)}$$

This is consistent with progressive high-temperature modification of the graphene shell, including wall thinning and increased permeability, which reduces the previously dominant diffusion barrier and enables more efficient product transport. The improved permeability facilitates the simultaneous progression of evaporation and reduction, thereby enhancing material removal. In addition, any CO_2_ formed locally during these carbothermic steps may further react with carbon at elevated temperatures, providing a secondary gasification pathway that could contribute to additional carbon loss and pore development. Under the present forming-gas conditions, however, this process is considered a secondary high-temperature contribution rather than the primary etching mechanism. Finally, in Phase 4 (up to ∼1100 °C), the etching process accelerates further, but enters a transport-limited regime again, governed primarily by the ability to rapidly evacuate gaseous reaction products (Zn and H_2_O). Sustaining an efficient removal of these species is essential to maintain the concentration gradients required for continuous etching.

Mechanical and mechanoelectrical characterization was conducted to assess how gas-phase template removal influences the functional performance of the resulting graphene-based aeromaterials. The cyclic compression tests (Figure 8a) revealed an increase in the apparent elastic modulus after the first loading cycle for all samples, consistent with structural settling or local rearrangement within the highly porous networks. Across the temperature range of 800–1000 °C, the stiffness of the dry-etched samples remained within the experimental scatter of the wet-chemically etched reference aeromaterials, indicating that the dry-chemical pathway preserves the intrinsic mechanical compliance of the underlying 3D architecture. Samples etched at 1100 °C constituted the only notable deviation, exhibiting a pronounced increase in stiffness. Considering the simultaneous SEM-observed wall thinning and microstructural irregularities at this temperature, this effect is not interpreted as global densification of the aeromaterial. Instead, it is more plausibly attributed to local thermally induced reorganization and consolidation of the load-bearing framework, for example at struts, junctions, or interflake contacts, which may reduce structural compliance despite increasing shell-level defects.^47^ However, fracture strength and brittle failure behavior were not quantified in the present study. Within the 800–1000 °C process window, these high-temperature effects do not translate into a pronounced loss of macroscopic mechanical compliance, whereas at 1100 °C clear signs of microstructural degradation become apparent.

**Figure 8:**
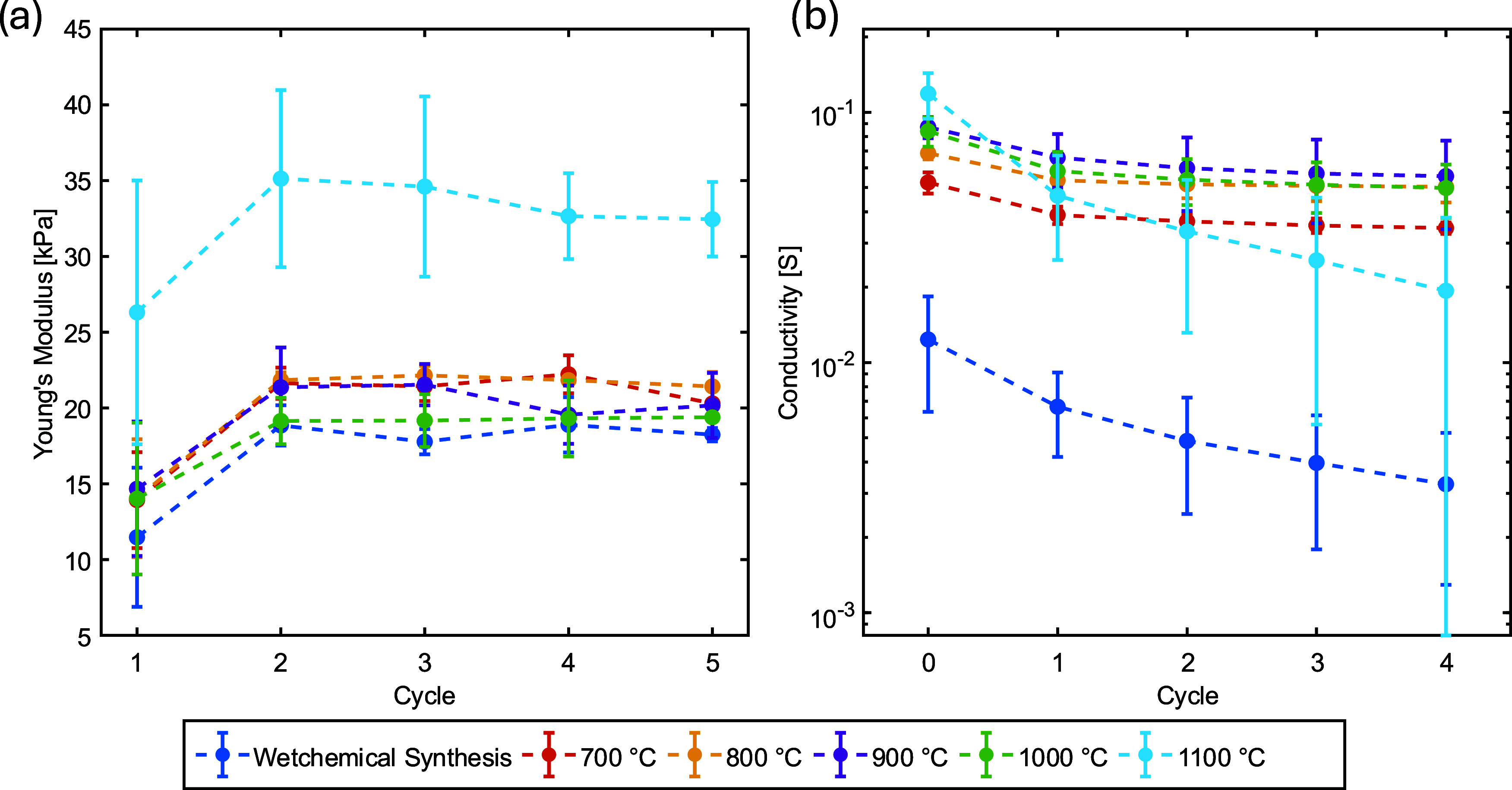
(a) Modulus of elasticity increases after initial compression. Qualitatively, all moduli of elasticity are of the same order of magnitude. 1100 °C is higher. (b) Comparison of conductivity values over five measurement cycles (Cycle 1–5) for different sample groups (Wet-Chemical Synthesis, 700–1100 °C). Wet-Chemical synthesized samples exhibit high variability and lower conductivity, while thermally treated groups (≥700 °C) show markedly higher, more stable, and reproducible conductivity across all cycles. Values are reported as mean ± SD (*n* = 3).

The results of the 30% compression experiments (Figure S8) corroborate this trend: dry-etched samples produced between 800 and 1000 °C displayed Young’s moduli comparable to those of the wet-etched reference material, whereas the reference group showed considerably larger variance. This reduced scatter suggests that the dry-chemical process yields mechanically more homogeneous aeromaterials, as evidenced by the narrower distribution of Young’s modulus values compared to the wet-etched references, likely due to the absence of liquid-induced deformation and stochastic damage during CPD.

The mechanoelectrical behavior closely paralleled the mechanical response. All dry-etched samples exhibited increased electrical conductivity under compressive strain, attributed to compression-induced network rearrangement, which reduces interstrut spacing, increases the effective graphene–graphene contact area, and activates additional percolation pathways within the conductive network. Upon unloading, the conductivity consistently returned to its baseline value, indicating predominantly elastic recovery of the conductive framework. Notably, the dry-etched aeromaterials showed substantially higher electrical conductivity than the wet-chemically etched references, up to approximately 4-fold at comparable geometry (Figure 8b). This enhancement is consistent with thermally induced removal of residual adsorbates and surface groups during the high-temperature etching step, which improves electrical coupling between the graphene flakes and reduces contact resistance. The samples treated at 1100 °C combined elevated stiffness with high electrical conductivity, further reflecting the interplay between structural densification and thermal cleaning at these temperatures.

## Conclusion

This work demonstrates that dry-chemical template removal in forming gas provides a robust and scalable route for fabricating ultralight, graphene-based aeromaterials from oxide templates. Using graphene-coated tetrapodal ZnO as a model system for oxide-templated 3D carbon architectures, it has been shown that gas-phase etching enables the production of hollow, highly porous microtube networks while largely preserving the macroscopic geometry and delicate microstructure of the underlying scaffold. Guided by the mechanistic insights obtained from the DSC and TGA analyses, a well-defined processing window (800–1000 °C) has been identified, enabling precise tuning of wall thinning, porosity, and defect formation, while preserving the macroscopic geometry and microstructural integrity of the aeromaterials.

Thermogravimetric and calorimetric analyses reveal a sequence of distinct temperature regimes that govern the dry-etching process: an initial desorption regime, followed by hydrogen-driven reduction of ZnO to metallic Zn, a subsequent transition toward vapor-transport-limited evaporation, and the onset of carbothermic reduction at higher temperatures. These mechanisms collectively explain the observed temperature dependence of the etching kinetics and the evolution of wall thinning, defect formation, and pore opening within the graphene shell. The resulting mechanistic framework is not restricted to the specific system studied here but is broadly relevant to hydrogen-assisted, gas-phase removal of reducible metal oxide templates.

Mechanical characterization under cyclic compression shows that, for process temperatures between 800 and 1000 °C, dry-etched aeromaterials retain stiffness comparable to that of wet-etched references, while exhibiting reduced sample-to-sample variability. This indicates that the absence of liquid-phase capillary forces and solvent-exchange steps favors mechanically uniform structures. Samples etched at 1100 °C display increased stiffness, consistent with partial densification and thermally induced microstructural degradation, manifested by wall thinning, pore opening, and local strut coalescence at very high temperatures. Mechanoelectrical measurements further demonstrate that the electrical conductivity of dry-etched samples can exceed that of wet-etched counterparts by up to approximately a factor of 4 at similar geometry, with stable, reversible responses under cyclic loading. These improvements are attributed to thermally induced removal of residual adsorbates and surface groups, as well as to microstructural changes that enhance internal conductive connectivity.

The dry-chemical approach inherently generates a zinc-containing vapor stream during template removal. As suggested by literature reports on hydrogen-based ZnO reduction in related systems, this metallic zinc could, in principle, be condensed, collected, and recycled into new feedstock, thereby closing the material loop and further improving the resource efficiency of the overall process chain. Implementing such zinc recovery concepts in future furnace designs would couple the advantages of gas-phase template removal with enhanced material circularity.

Overall, the presented study establishes gas-phase template removal in forming gas as a technically and ecologically attractive alternative to conventional wet-chemical template removal for oxide-templated carbon aeromaterials. The approach combines controllable microstructural tuning, favorable mechanical and electrical performance, while being inherently compatible with scalable and automated furnace processing. As such, it provides a promising basis for the application-specific design and sustainable large-scale fabrication of ultralight, electrically conductive aeromaterials for use in sensing, soft actuation, lightweight structural components, and for filtration and membrane technologies.

## Supplementary Material



## Notes

Photographs and SEM images of dry-etched aerographene samples at different synthesis temperatures (Figures S1–S3); porosity and effective density analysis (Figure S4); geometrical evolution and volumetric shrinkage during processing (Figures S5 and S6); graphene mass retention after etching (Figure S7); and additional mechanical characterization data (Figure S8) (PDF)
