# Supplementary material for: Dry-Etched Oxide Templates Enable Scalable and Waste-Free Synthesis of Graphene-Based Aeromaterials
Source: ACS Appl Eng Mater. 2026 May 6;4(5):2444–55. doi: 10.1021/acsaenm.6c00181 (PMC13200186; doi:10.1021/acsaenm.6c00181)
Supplement: Supplementary file 1 [file em6c00181_si_001.pdf]

Suporting Information

Dry Etched Oxide Templates Enable Scalable and Waste-Free Synthesis of Graphene-Based Aeromaterials

Morten Möller*, K. Meurisch, J.-O. Stern**, A. Reimers, F. Schütt, Sören Kaps, Rainer Adelung

Functional Nanomaterials, Department of Materials Science, Kiel University, Kaiserstr. 2, 24143 Kiel, Germany

Email: *mom@tf.uni-kiel.de, **jost@tf.uni-kiel.de

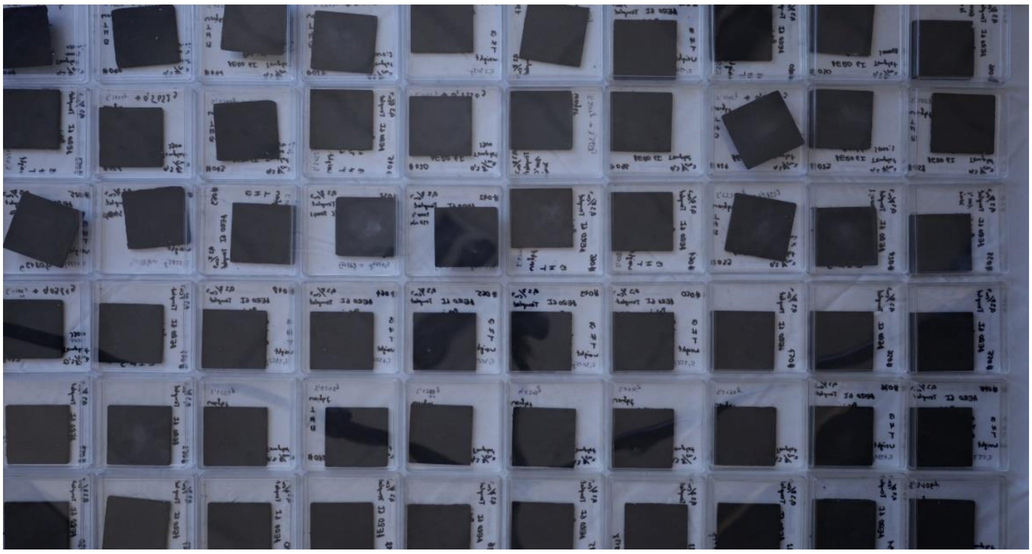

Figure S2: *Overview of 60 out of 250 aerographene samples fabricated in the 68 × 68 × 4 mm plate format.*

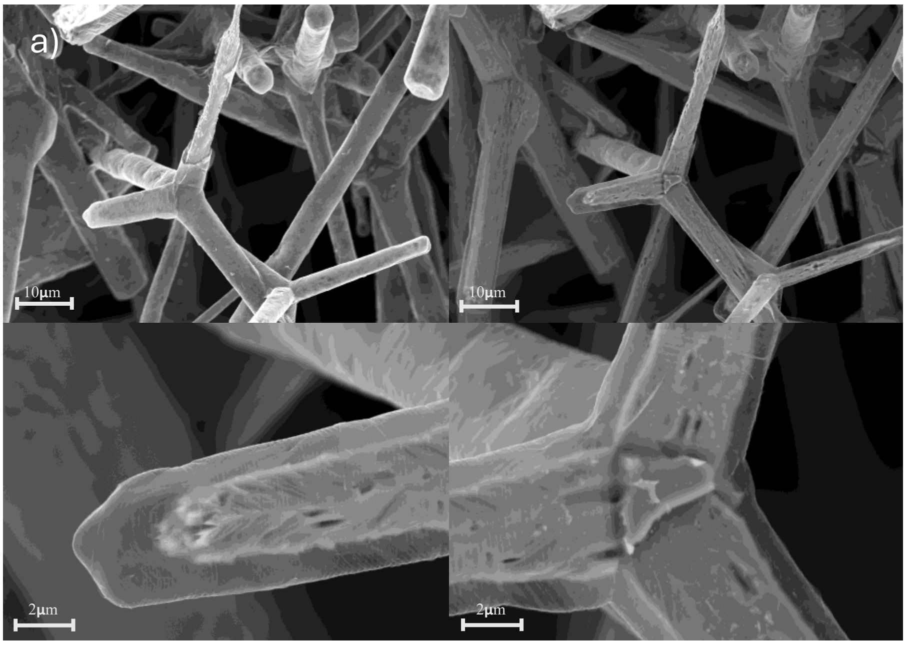

Figure S1*: SEM images of a tetrapod that was dry-etched at 600 °C for 5 hours in a forming gas atmosphere. Incomplete etching is visible. From top to bottom: An image of a tetrapod incompletely coated with graphene; the same tetrapod at increased acceleration voltage of the SEM. The graphene layer is transparent; close-up of one arm of the same tetrapod; close-up of the core of the same tetrapod.*

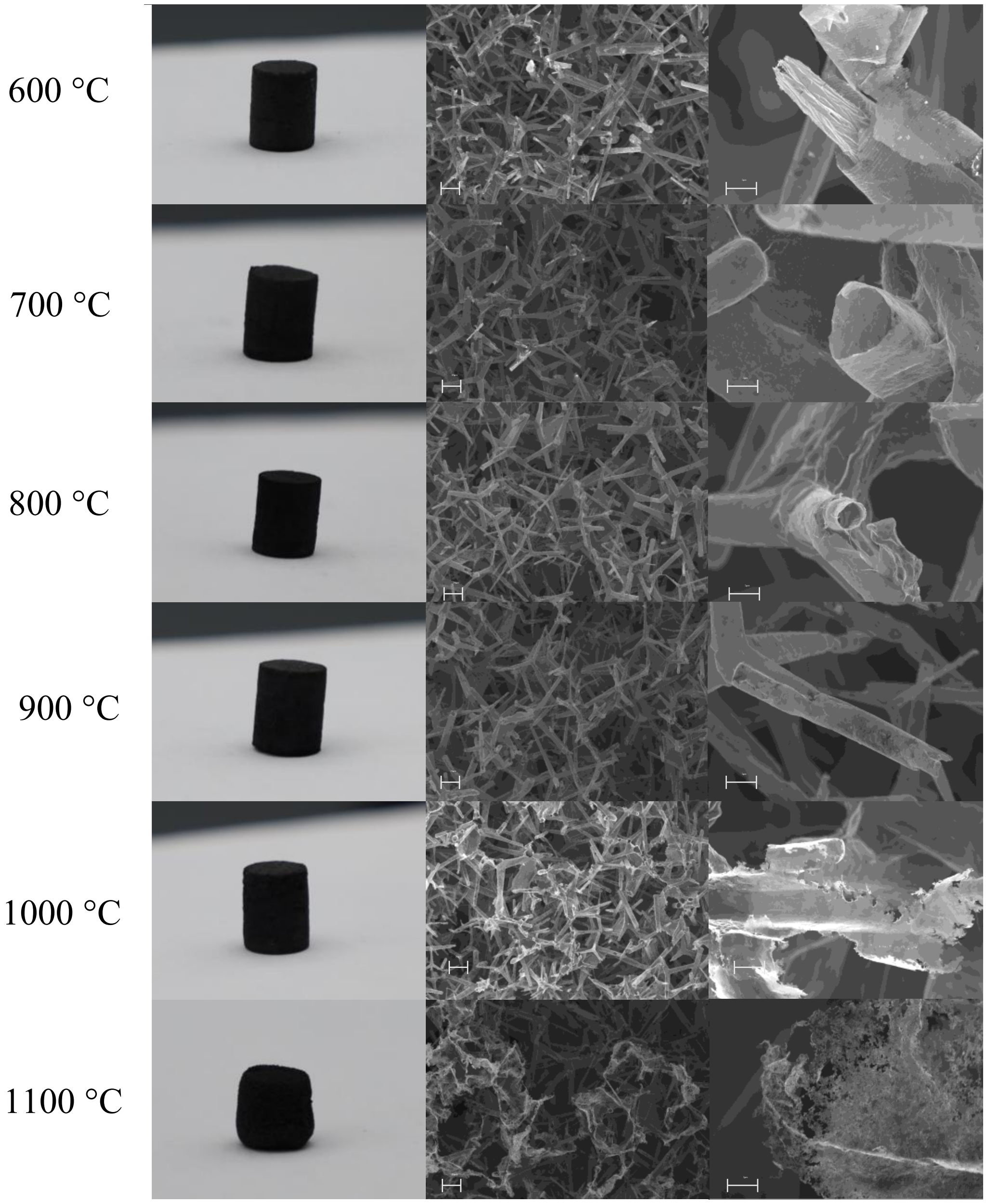

Figure S3: *Photos and REM images of cylindrical Ø6mm x 6mm high aerograph samples corresponding to the synthesis temperature with associated SEM images*

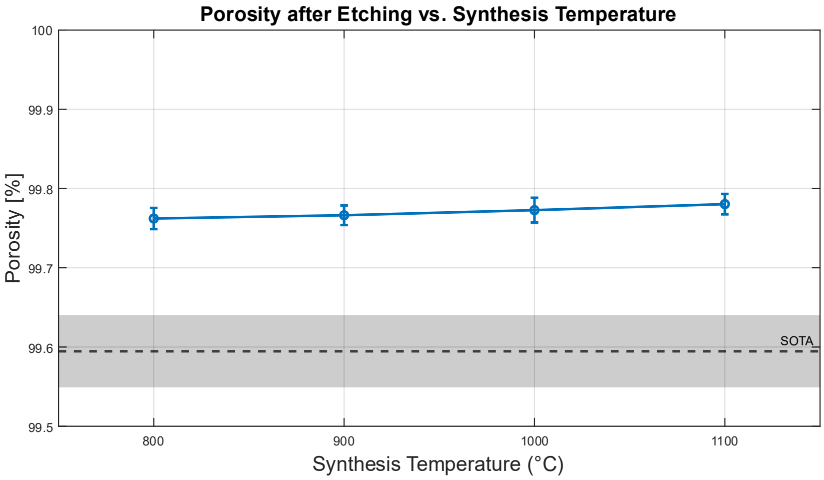

Figure S4: *The resulting porosity of the dry-etched samples was ~99.8 % (mean ± sd, n = 3), underlining the effectiveness of the etching process in producing an almost entirely open porous network.*

Geometrical Changes During Processing

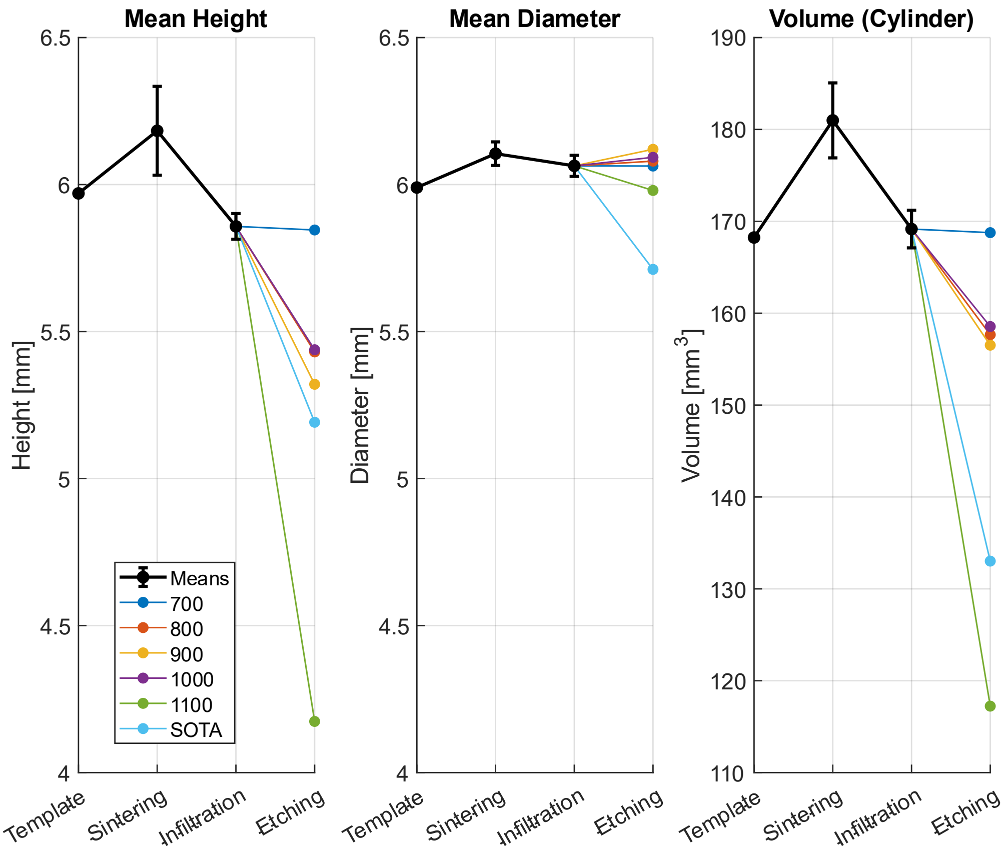

Figure S5: *Evolution of height, diameter, and volume of a cylindrical sample over four processing steps. Mean values with error bars are shown up to the infiltration step; individual sample values are displayed after etching. (SOTA = Wetchemical Synthesis). Values represent mean values of n = 5 wet-etched samples and n = 3 dry-etched samples per temperature.*

Figure S5 illustrates the average evolution of sample geometry (height and diameter) throughout processing steps (sintering, infiltration, etching). Slight expansion in both height and diameter was observed after sintering. Following infiltration, moderate shrinkage occurred, likely due to solvent evaporation and drying of the graphene dispersion.

From the infiltration step onward, the individual geometrical changes for each sample are shown. A pronounced volume reduction was detected after the etching step, attributed to the removal of the ZnO scaffold structure.

Shrinkage Behavior

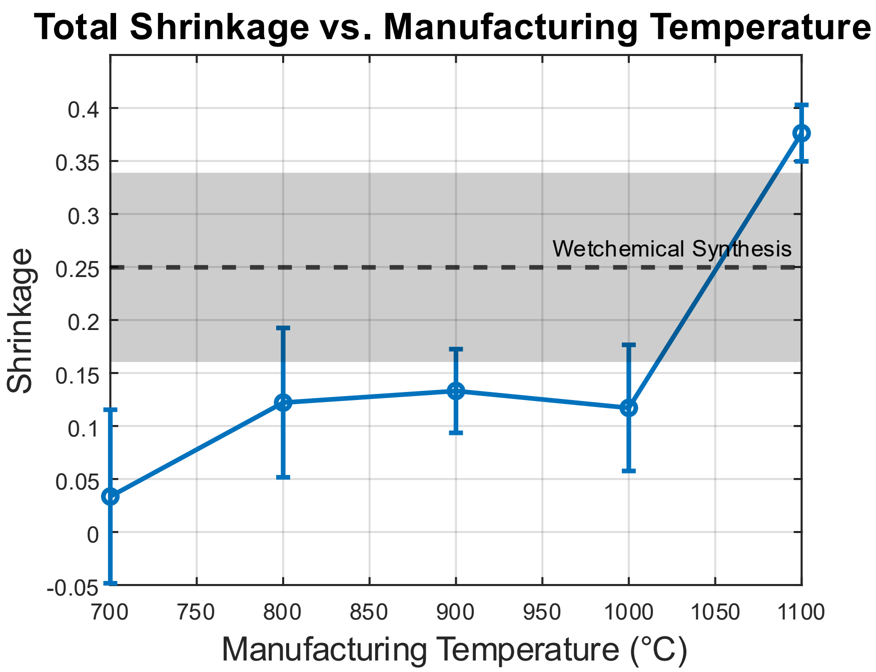

Figure S6: *Total volumetric shrinkage of the samples as a function of processing temperature, including comparison with the SOTA method. Values represent mean values of n = 5 wet-etched samples and n = 3 dry-etched samples per temperature.*

Figure S6 summarizes the total volumetric shrinkage as a function of process temperature. Between 800 °C and 1000 °C, an average shrinkage of ~12.5 % was observed (±7.5 %). In comparison, the SOTA reference exhibited a higher average shrinkage of 25 % (±10 %).

Samples processed at 1100 °C showed significantly higher shrinkage, which may be due to thermal degradation or structural instability at elevated temperatures. The ratio of volume loss to mass loss suggests that not only pore compaction occurred, but also partial removal of graphene material during the etching process.

Mass Retention and Graphene Recovery

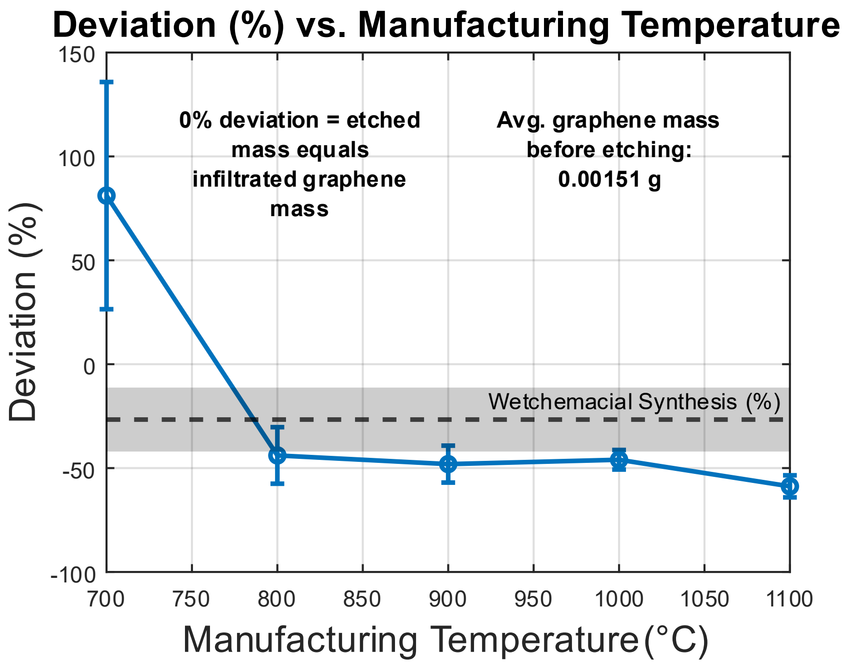

Figure S7: *Percentage deviation of the residual mass after etching from the initially introduced graphene mass as a function of processing temperature. Results from the SOTA reference process are included for comparison. Values represent mean values of n = 5 wet-etched samples and n = 3 dry-etched samples per temperature.*

As shown in Supplementary **Figure S7**, the sample processed at 700 °C exhibits a significantly higher residual mass compared to the other temperature groups, indicating incomplete ZnO removal. For this reason, data from the 700 °C group were excluded from subsequent average-based analyses.

The introduced graphene mass was determined from the mass difference between the infiltrated and sintered state. The amount of graphene remaining after etching was then compared to this initial value. **Figure S7** displays the relative graphene mass retained after etching as a function of process temperature.

All samples show at least a 25 % mass loss. The wet-chemical reference process (SOTA) yielded the lowest average loss (25 %), while the dry-chemically etched samples (800–1000 °C) consistently showed a ~45 % loss. Notably, the variance in graphene retention decreased with increasing temperature, suggesting either a more stable graphene network or a temperature-dependent change in pore morphology.

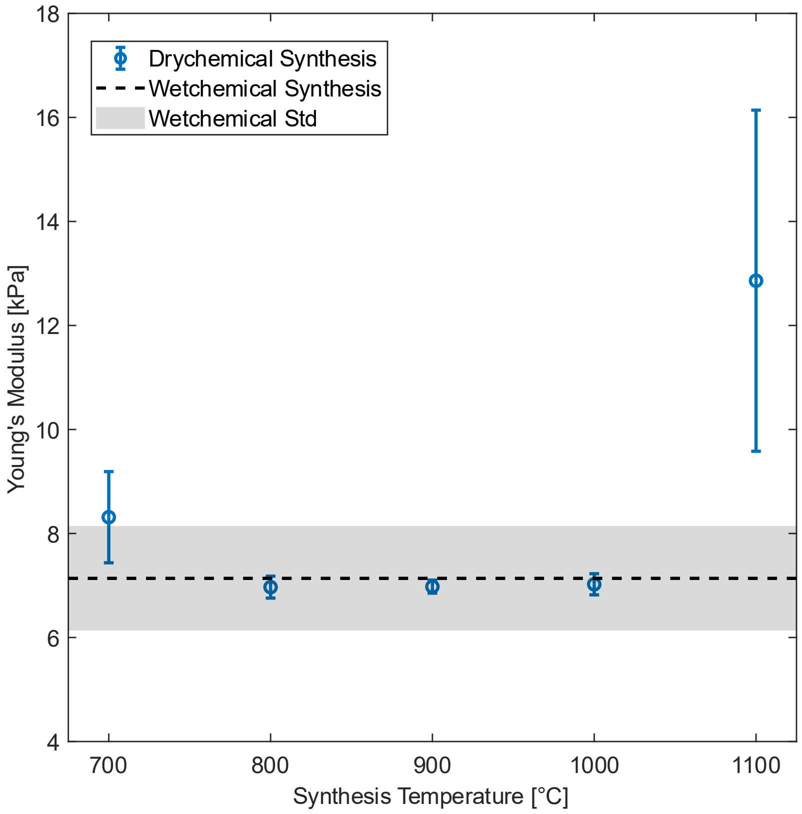

Figure S8: *Young's Modulus over Synthesis temperature after 30% compression. Values are reported as mean ± SD (n = 3)*
